# Correction: Differential regulation of hair cell actin cytoskeleton mediated by SRF and MRTFB

**DOI:** 10.7554/eLife.112679

**Published:** 2026-07-27

**Authors:** Ling-Yun Zhou, Chen-Xi Jin, Wen-Xiao Wang, Lei Song, Jung-Bum Shin, Ting-Ting Du, Hao Wu

**Keywords:** Mouse

Zhou L-Y, Jin C-X, Wang W-X, Song L, Shin J-B, Du T-T, Wu H. 2023. Differential regulation of hair cell actin cytoskeleton mediated by SRF and MRTFB. *eLife* **12**:e90155. doi: 10.7554/eLife.90155.

Published 20 November 2023

The bottom-left image of Figure 2D is a scanning electron microscopy image of IHC hair bundles from P10 Srf cKO mice, whereas the third row, second column image of Figure 9I is a scanning electron microscopy image of IHC hair bundles from P11 Srf cKO mice injected with the control virus (AAV-GFP). The two images closely resembled each other in overall morphology. During final assembly of the figures, while handling a large number of scanning electron microscopy image files to select representative ones, we inadvertently replaced the bottom-left image of Figure 2D with a duplicate of the third row, second column image from Figure 9I. This occurred because the cropped sub-images were not systematically named after cropping and were not cross-referenced with the original file names, leading us to drag the wrong file into the figure. Moreover, the mistakenly used image coincidentally displayed the characteristic Srf cKO hair bundle phenotype at P10, making it appear consistent with the expected result during our initial quality check.

We have now corrected the figure with the proper image (see below). This correction does not affect the interpretation or conclusions of the paper. The quantification statistics presented in Figure 2E were derived from the bottom-right image of Figure 2D, not the bottom-left image. Furthermore, the corrected image remains fully consistent with the original findings and does not alter any quantitative or qualitative results.

The corrected Figure 2 (lower left image corrected in panel D) is shown here:

**Figure 1:**
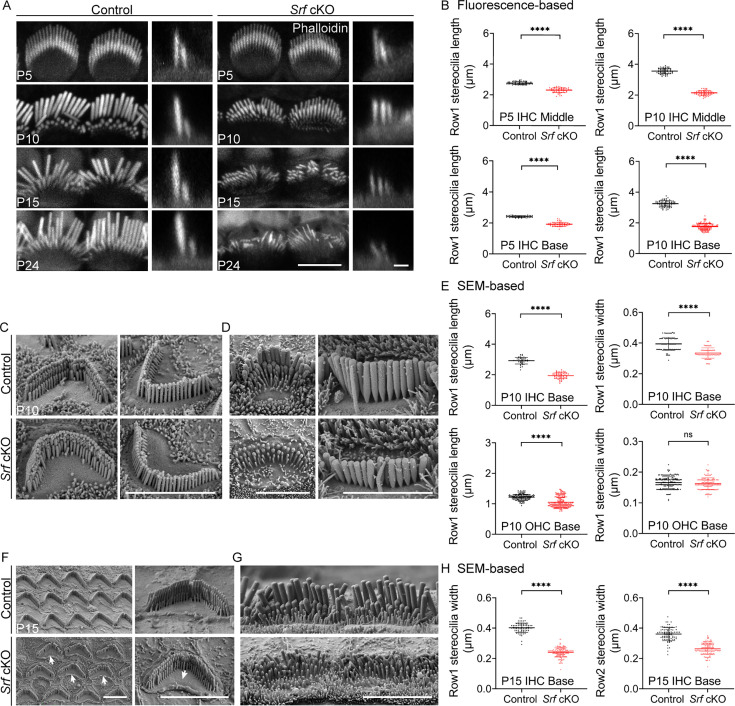


The originally published Figure 2 is shown for reference:

**Figure 2:**
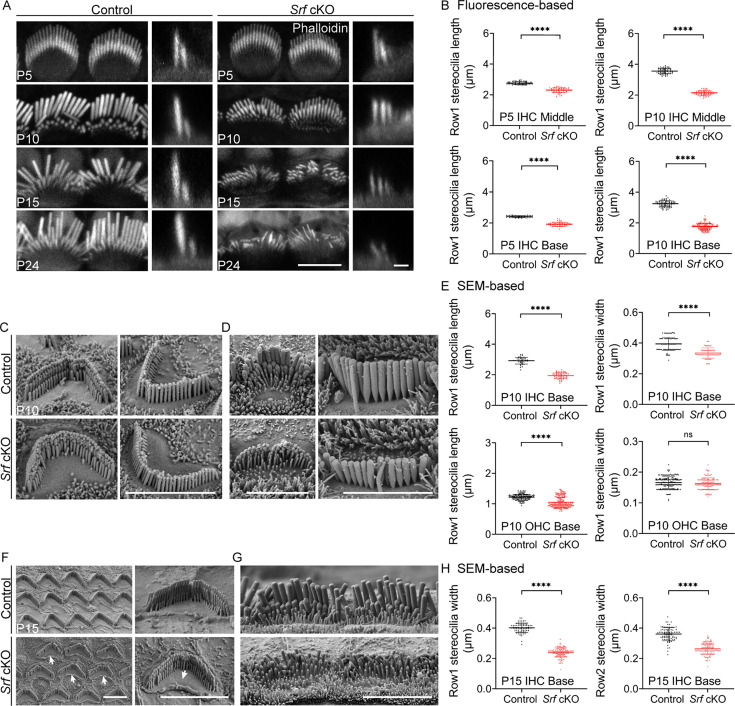


The article has been corrected accordingly. The original bottom-left image of Figure 2D is available as Source data 1. The original third row, second column image of Figure 9I is available as Source data 2.

## Additional files

Source data 1. Original bottom-left image of Figure 2D.

Source data 2. Original third row, second column image of Figure 9I.

